# Monitoring the impact of COVID-19 in France on cancer care: a differentiated impact

**DOI:** 10.1038/s41598-022-07984-w

**Published:** 2022-03-10

**Authors:** Christine Le Bihan Benjamin, Julien-Aymeric Simonnet, Mathieu Rocchi, Inès Khati, Estelle Ménard, Emilie Houas-Bernat, Jean-Baptiste Méric, Philippe-Jean Bousquet

**Affiliations:** 1 Health Data and Assessment Department, Survey Data Science and Assessment Division, National Cancer Institute, 52 Avenue André Morizet, 92100 Boulogne-Billancourt, France; 2 Care Paths Organization Department, Public Health Division, National Cancer Institute, 52 Avenue André Morizet, 92100 Boulogne-Billancourt, France; 3 Survey Data Science and Assessment Division, National Cancer Institute, 52 Avenue André Morizet, 92100 Boulogne-Billancourt, France; 4 Public Health Division, National Cancer Institute, 52 Avenue André Morizet, 92100 Boulogne-Billancourt, France; 5 Aix Marseille University, INSERM, IRD, Economics and Social Sciences Applied To Health and Analysis of Medical Information (SESSTIM), 27 Bd Jean Moulin, 13005, Marseille, France

**Keywords:** Cancer, Health care, Oncology

## Abstract

The COVID-19 pandemic has had a substantial and lasting impact on care provision, particularly in the field of cancer care. National steering has helped monitor the health situation and adapt the provision and organisation of care. Based on data from the French administrative healthcare database (SNDS) on the entire French population (67 million people), screening, diagnostic and therapeutic activity was monitored and compared 2019 on a monthly basis. A noteworthy decline in all activities (with the exception of chemotherapy) was observed during the first lockdown in France. Over the months that followed, this activity returned to normal but did not make up for the shortfall from the first lockdown. Finally, during the lockdown in late 2020, cancer care activity was conserved. In brief, in 2020, the number of mammograms decreased by 10% (− 492,500 procedures), digestive endoscopies by 19% (− 648,500), and cancer-related excision by 6% (− 23,000 surgical procedures). Hospital radiotherapy activity was down 3.8% (− 4400 patients) and that in private practice was down 1.4% (− 1600 patients). Chemotherapy activity increased by 2.2% (7200 patients), however. To summarize, COVID-19 had a very substantial impact during the first lockdown. Safeguarding cancer care activity helped limit this impact over the months that followed, but the situation remains uncertain. Further studies on the medium- and long-term impact on individuals (survival, recurrence, after-effects) will be conducted.

## Introduction

Since early 2020, the world has been faced with a pandemic caused by SARS-Cov-2. In terms of health, this has resulted in more or less stringent measures, and restrictions up to and including full lockdown. More precisely, France was under lockdown from 17 March to 11 May 2020, with a travel ban, the closure of so-called non-essential retail and businesses and recreational facilities, and extensive remote working. A second lockdown took place from 28 October to 15 December 2020, which was less restrictive than the first (schools stayed open, and a limited number of businesses continued to operate). Following this lockdown, the entire country was placed under curfew. In March 2021, further measures were gradually introduced to supplement the measures in place, starting with some French departments and then extended throughout the country.

In the field of cancer care, some measures were specifically drafted with in particular a number of guidelines and tools being issued for healthcare professionals by the French Ministry for Health and the French National Cancer Institute (INCa), ranging from adaptation of medical practices in crisis situations, to the resumption of activity, along with the prioritisation of cancer patients for vaccination^1–5^ https://www.e-cancer.fr/Professionnels-de-sante/Coronavirus-COVID-19). Furthermore, during the first lockdown, invitations for organised screening programmes (breast, colorectal, cervical) were suspended^6^, and a number of treatments directly linked with cancer care deferred (surgery, cancer-related hospitalisation, etc.). Similar initiatives have been adopted in most Western countries, repositioning screening with regard to risks due to COVID-19 and in the aim of adapting the healthcare system^7–9^.

In view of the risk of missing a diagnosis or of prolonging times to treatment for a substantial number of people, the measures in relation to screening were not renewed during subsequent lockdown periods, despite intensive care unit occupancy being on a par with or exceeding usual capacity. Indeed, a number of studies suggest a reduction in survival associated with increased waiting times to undergo screening^10–12^, diagnosis, or treatment^13–15^. This had led the health authorities to propose new strategies, prioritise cancer patients, and take action in the uncertainty.

In this context, the French National Cancer Institute has set up, with support from the French Ministry for Health, a national steering and monitoring committee in concert with major national and regional stakeholders in cancer care and user representatives. It is organised in a regional structure via local and regional steering committees, helping pass on alerts and important information, and report, on a national level, innovative and exemplary organisations along with issues encountered on a regional level.

The Institute has also developed activity monitoring and steering scorecards aimed at national, regional and local stakeholders. They are intended to monitor prevention, screening, and care activity in hospital and non-hospital settings.

The purpose of this study is to present the cancer care activity monitoring and steering scorecards, estimate the impact of the health crisis due to COVID-19, and present the measures adopted to limit their effects.

## Methodology

### Data source

Several data sources were used. For non-hospital activity, data from the French administrative healthcare database (SNDS), medico-administrative data covering the entire French population^16^ were used. These data are updated on a monthly basis.

For hospital activity, the study relied on activity data recorded by all French hospital facilities (Medicalised information system programme) for all inpatients and outpatients. Since the pandemic, these data have been updated on a weekly basis. Although they are also found in the SNDS, these data are processed on the secure ATIH (French agency for information on hospital management) due to quicker availability.

Three months after the activity completion date, these two data sources are sufficiently exhaustive for processing purposes.

### Data

Activity monitoring concerns the analysis of medical procedures linked with cancer care for diagnosis, screening, or treatment (excision, chemotherapy, radiotherapy). Lines were selected according to the public health policy in France. For diagnosis, the lines of analysis are upper and lower digestive tract endoscopies, bronchial and ENT endoscopies (fibroscopies), prostate biopsies as per the CCAM (Common classification of medical procedures) list.

For screening, the lines are mammograms under the organised breast cancer screening programme or outside this screening programme, faecal blood screening tests for the organised colorectal cancer screening programme, cytological analyses or human papillomavirus detection for organised cervical screening or outside the organised cervical screening programme as per the different lists concerned (CCAM, NABM – list of medical pathology procedures, and NGAP – general list of professional procedures).

For cancer care-related excisions, the lines of analysis concern the 6 categories of cancer sites for which activity is subject to authorisation^17^ and to minimum activity thresholds in France: digestive tract (stomach, liver, pancreas, colorectal), gynaecological (ovarian), breast, chest, urological, and ENT (ear, nose, throat) and maxillofacial cancers (CCAM). Oesophageal cancers are excluded from digestive tract, chest and ENT cancers, and presented separately for a better understanding. Stays with cancer removal surgery are identified by coding cancer as the main diagnosis and a surgical removal procedure. Thus, only histologically confirmed cancers should be counted.

For chemotherapy, the data used were from hospital admissions specifying a primary diagnostic code Z511 (ICD 10 – International Classification of Diseases); and, for radiotherapy, hospital admissions specifying a primary diagnostic code Z5101 (ICD 10), and private practice CCAM radiation procedures.

### Analyses

Monthly comparisons (number and percent) of care consumption for the years 2019 and 2020 were made to account for seasonal factors in care activity.

### Ethic

All methods were carried out in accordance with relevant guidelines and regulations. Data were pseudonymized prior to performing analyses Access to SNDS and PMSI data is subject to authorisation from CNIL (French data protection authority)—decree of 26 December 2016 No. 2016–1871.

## Results

The study covered the entire French population, i.e. 67 million people, sex-ratio 0,93 and mean age 42,1 years.

### Diagnosis, screening (Table 1, Fig. 1)

**Table 1:** Diagnosis and screening.

	January	February	March	April	May	June	July	August	September	October	November	December	Total
**Digestive tract endoscopies**													
2019	**308,959**	**286,816**	**308,816**	**304,647**	**298,963**	**289,361**	**284,372**	**170,407**	**290,696**	**318,586**	**282,356**	**253,945**	3,397,924
2020	**308,279**	**281,211**	**174,032**	**53,294**	**141,927**	**262,496**	**270,006**	**175,477**	**294,410**	**289,393**	**254,132**	**244,734**	2,749,391
2021	**279,495**	**263,955**	**286,928**										830,378
*Difference 2019/2020*	− 680	− 5605	− 134,784	− 251,353	− 157,036	− 26,865	− 14,366	5070	3714	− 29,193	− 28,224	− 9211	− 648,533
*Percent 2019/2020*	− 0.2	− 2.0	− 43.6	− 82.5	− 52.5	− 9.3	− 5.1	3.0	1.3	− 9.2	− 10.0	− 3.6	− 19.1
*Difference 2019/2021*	− 29,464	− 22,861	− 21,888										− 74,213
*Percent 2019 − 2021*	− 9.5	− 8.0	− 7.1										
**Bronchial and ENT endoscopies**													
2019	**107,085**	**95,901**	**106,923**	**103,516**	**101,351**	**96,611**	**98,820**	**66,621**	**99,083**	**106,101**	**95,484**	**88,893**	1,166,389
2020	**104,733**	**97,579**	**64,651**	**28,750**	**60,831**	**92,028**	**85,333**	**60,821**	**93,928**	**87,278**	**85,568**	**81,283**	942,783
2021	**87,914**	**82,669**	**94,018**										264,601
*Difference 2019/2020*	− 2352	1678	− 42,272	− 74,766	− 40,520	− 4583	− 13,487	− 5800	− 5155	− 18,823	− 9916	− 7610	− 223,606
*Percent 2019/2020*	− 2.2	1.7	− 39.5	− 72.2	− 40.0	− 4.7	− 13.6	− 8.7	− 5.2	− 17.7	− 10.4	− 8.6	− 19.2
*Difference 2019/2021*	− 19,171	− 13,232	− 12,905										− 45,308
*Percent 2019–2021*	− 17.9	− 13.8	− 12.1										
**Mammograms**													
2019	**473,347**	**421,823**	**461,522**	**445,034**	**442,258**	**423,883**	**404,342**	**284,033**	**449,133**	**494,668**	**445,629**	**386,486**	5,132,158
2020	**478,150**	**437,774**	**254,122**	**66,597**	**278,076**	**455,482**	**406,343**	**316,109**	**504,807**	**508,138**	**495,773**	**438,251**	4,639,622
2021	**474,674**	**445,114**	**523,430**										1,443,218
*Difference 2019/2020*	4803	15,951	− 207,400	− 378,437	− 164,182	31,599	2001	32,076	55,674	13,470	50,144	51,765	− 492,536
*Percent 2019/2020*	1.0	3.8	− 44.9	− 85.0	− 37.1	7.5	0.5	11.3	12.4	2.7	11.3	13.4	− 9.6
*Difference 2019/2021*	1327	23,291	61,908										86,526
*Percent 2019 − 2021*	0.3	5.5	13.4										
**Colorectal screening**													
2019	**212,938**	**199,831**	**200,709**	**160,751**	**116,359**	**92,929**	**72,041**	**68,759**	**139,420**	**225,007**	**273,084**	**240,014**	2,001,842
2020	**293,675**	**310,605**	**206,161**	**12,976**	**68,865**	**169,754**	**212,947**	**215,809**	**325,936**	**353,617**	**324,902**	**305,949**	2,801,196
2021	**288,421**	**276,819**	**406,604**										971,844
*Difference 2019/2020*	80,737	110,774	5452	− 147,775	− 47,494	76,825	140,906	147,050	186,516	128,610	51,818	65,935	799,354
*Percent 2019/2020*	37.9	55.4	2.7	− 91.9	− 40.8	82.7	195.6	213.9	133.8	57.2	19.0	27.5	39.9
*Difference 2019/2021*	75,483	76,988	205,895										358,366
*Percent 2019 − 2021*	35.4	38.5	102.6										
**Cervix screening (HPV and cytopathology)**													
2019	**411,678**	**378,100**	**416,419**	**399,230**	**397,281**	**377,125**	**370,493**	**242,953**	**398,372**	**424,446**	**369,701**	**337,462**	4,523,260
2020	**410,798**	**377,973**	**252,407**	**80,142**	**260,984**	**433,787**	**366,413**	**259,785**	**430,692**	**414,737**	**393,093**	**342,051**	4,022,862
2021	**369,688**	**342,946**	**360,992**										1,073,626
*Difference 2019/2020*	− 880	− 127	− 164,012	− 319,088	− 136,297	56,662	− 4080	16,832	32,320	− 9709	23,392	4589	− 500,398
*Percent 2019/2020*	− 0.2	0.0	− 39.4	− 79.9	− 34.3	15.0	− 1.1	6.9	8.1	− 2.3	6.3	1.4	− 11.1
*Difference 2019/2021*	− 41,990	− 35,154	− 55,427										− 132,571
*Percent 2019 − 2021*	− 10.2	− 9.3	− 13.3										
**Prostatic biopsies**													
2019	**9562**	**8447**	**9075**	**8810**	**8675**	**8247**	**7826**	**5120**	**9099**	**9004**	**8102**	**6853**	98,820
2020	**10,151**	**8560**	**6217**	**2975**	**6601**	**9176**	**7549**	**5042**	**9201**	**8621**	**8673**	**7451**	90,217
2021	**9390**	**8251**	**8989**										26,630
*Difference 2019/2020*	589	113	− 2858	− 5835	− 2074	929	− 277	− 78	102	− 383	571	598	− 8603
*Percent 2019/2020*	6.2	1.3	− 31.5	− 66.2	− 23.9	11.3	− 3.5	− 1.5	1.1	− 4.3	7.0	8.7	− 8.7
*Difference 2019/2021*	− 172	− 196	− 86										− 454
*Percent 2019 − 2021*	− 1.8	− 2.3	− 0.9										

Number of screening and diagnostic acts observed are in [bold].

*HPV* human papilloma viridae.

**Figure 1:**
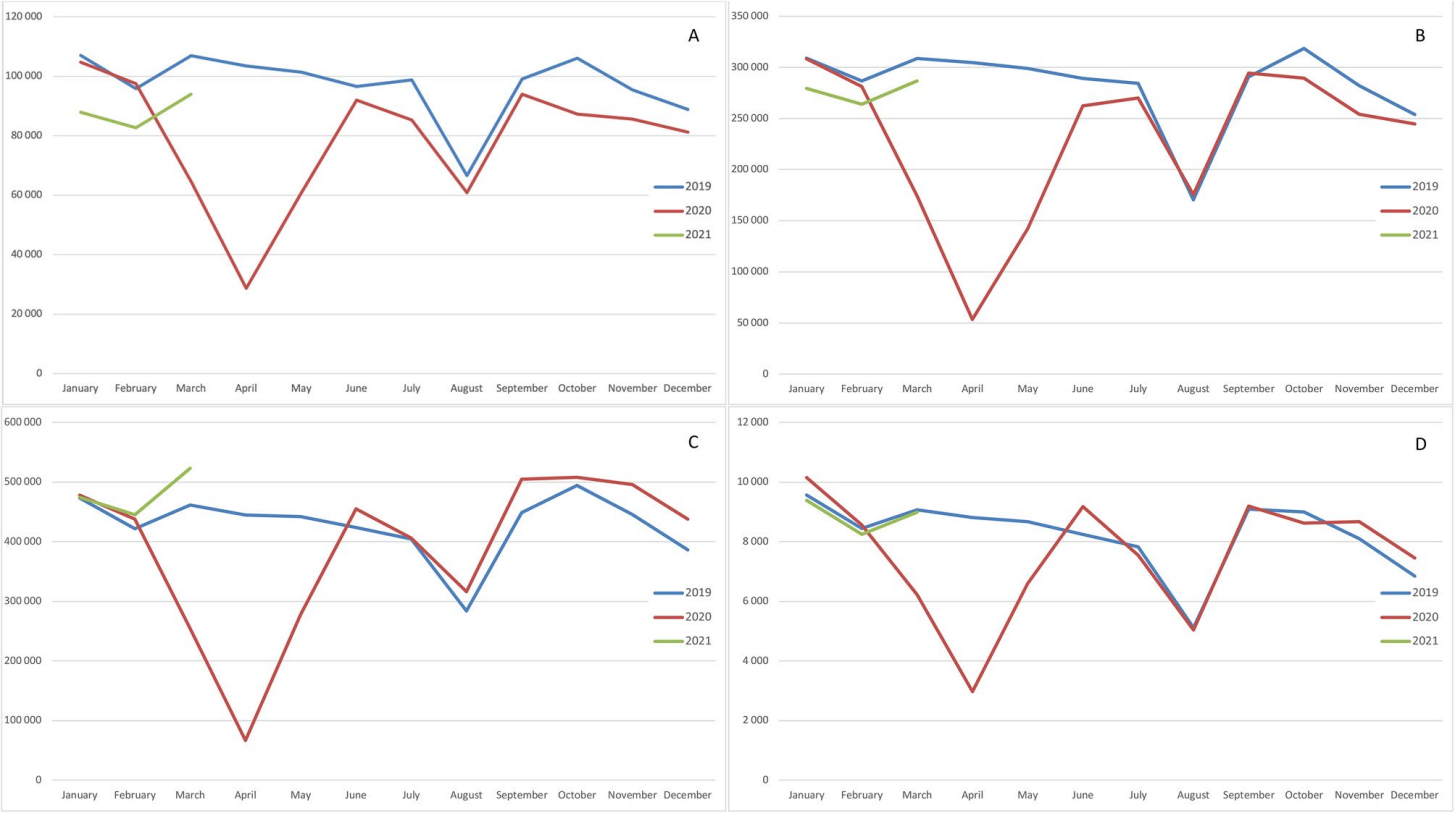
Screening and diagnosis trends. (**A**) Fibroscopies (ear, nose and throat—ENT and lung). (**B**) Endoscopies (Digestive track). (**C**) Mammograms. (**D**) Prostatic biopsies.

Diagnostic and screening activity was substantially impacted by the first lockdown.

For digestive tract endoscopies, the shortfall was − 43.6% (− 134,784 procedures) in March, − 82.5% (− 251,353) in April, − 52.5% (− 157,036) in May, − 9.3% (− 26,865) in June, and − 5.1% (− 14,366) in July. Over the full year, a shortfall of -19.1% (− 648,533) remained.

For mammograms, the shortfall was − 44.9% (− 207,400 procedures) in March, -85% (− 378,437) in April, − 37.1% (− 164,182) in May. Despite there being no further decreases in activity from June, a shortfall of − 9.6% (− 492,536) remained over the full year.

Similar findings were observed for bronchial and ENT fibroscopies and prostate biopsies. An overall increased in colorectal screening and decrease in cervical screening (HPV test and cytopathology) were observed between 2019 and 2020. (see Table 1 and Fig. 1).

### Surgery (excision) (Table 2)

**Table 2:** Surgery (excision).

	January	February	March	April	May	June	July	August	September	October	November	December	Total
**Oesophagus**													
2019	**67**	**90**	**99**	**111**	**108**	**107**	**124**	**93**	**81**	**115**	**90**	**125**	1210
2020	**89**	**83**	**99**	**65**	**65**	**97**	**121**	**66**	**81**	**95**	**87**	**90**	1038
2021	**83**	**80**	**91**										254
*Difference 2019/2020*	22	− 7	0	− 46	− 43	− 10	− 3	− 27	0	− 20	− 3	− 35	− 172
*percent 2019/2020*	32.8	− 7.8	0.0	− 41.4	− 39.8	− 9.3	− 2.4	− 29.0	0.0	− 17.4	− 3.3	− 28.0	− 14.2
*Difference 2019/2021*	16	− 10	− 8										− 2
*Percent 2019/2021*	23.9	− 11.1	− 8.1										
**Stomach**													
2019	**252**	**254**	**297**	**270**	**262**	**266**	**296**	**216**	**248**	**312**	**270**	**270**	3213
2020	**244**	**271**	**276**	**193**	**217**	**266**	**278**	**173**	**204**	**261**	**253**	**283**	2919
2021	**232**	**224**	**231**										687
*Difference 2019/2020*	− 8	17	− 21	− 77	− 45	0	− 18	− 43	− 44	− 51	− 17	13	− 294
*Percent 2019/2020*	− 3.2	6.7	− 7.1	− 28.5	− 17.2	0.0	− 6.1	− 19.9	− 17.7	− 16.3	− 6.3	4.8	− 9.2
*Difference 2019/2021*	− 20	− 30	− 66										− 116
*Percent 2019/2021*	− 7.9	− 11.8	− 22.2										
**Liver**													
2019	**544**	**595**	**654**	**618**	**585**	**618**	**605**	**422**	**576**	**667**	**545**	**564**	6993
2020	**517**	**576**	**628**	**450**	**478**	**583**	**577**	**424**	**523**	**596**	**546**	**610**	6508
2021	**502**	**562**	**601**										1665
*Difference 2019/2020*	− 27	− 19	− 26	− 168	− 107	− 35	− 28	2	− 53	− 71	1	46	− 485
*Percent 2019/2020*	− 5.0	− 3.2	− 4.0	− 27.2	− 18.3	− 5.7	− 4.6	0.5	− 9.2	− 10.6	0.2	8.2	− 6.9
*Difference 2019/2021*	− 42	− 33	− 53										− 128
*Percent 2019/2021*	− 7.7	− 5.5	− 8.1										
**Pancreas**													
2019	**280**	**337**	**364**	**381**	**323**	**305**	**371**	**294**	**275**	**347**	**300**	**397**	3974
2020	**308**	**329**	**355**	**248**	**256**	**333**	**389**	**258**	**300**	**362**	**301**	**371**	3810
2021	**265**	**366**	**345**										976
*Difference 2019/2020*	28	− 8	− 9	− 133	− 67	28	18	− 36	25	15	1	− 26	− 164
*Percent 2019/2020*	10.0	− 2.4	− 2.5	− 34.9	− 20.7	9.2	4.9	− 12.2	9.1	4.3	0.3	− 6.5	− 4.1
*Difference 2019/2021*	− 15	29	− 19										− 5
*Percent 2019/2021*	− 5.4	8.6	− 5.2										
**Colon − Rectum**													
2019	**2645**	**2907**	**3139**	**3200**	**3163**	**2898**	**3453**	**2571**	**2624**	**3097**	**2732**	**3018**	35,447
2020	**2509**	**2794**	**3344**	**2298**	**2225**	**2580**	**3032**	**2529**	**2672**	**2912**	**2839**	**3138**	32,872
2021	**2558**	**2692**	**2961**										8211
difference 2019/2020	− 136	− 113	205	− 902	− 938	− 318	− 421	− 42	48	− 185	107	120	− 2575
percent 2019/2020	− 5.1	− 3.9	6.5	− 28.2	− 29.7	− 11.0	− 12.2	− 1.6	1.8	− 6.0	3.9	4.0	− 7.3
difference 2019/2021	− 87	− 215	− 178										− 480
percent 2019/2021	− 3.3	− 7.4	− 5.7										
**ENT + Maxillofacial**													
2019	**1968**	**1836**	**2109**	**1956**	**2069**	**1896**	**2062**	**1545**	**1908**	**2071**	**2002**	**2026**	23,448
2020	**2040**	**1915**	**1938**	**1402**	**1538**	**1683**	**1813**	**1469**	**1913**	**1916**	**1872**	**1903**	21,402
2021	**1865**	**1811**	**1883**										5559
*Difference 2019/2020*	72	79	− 171	− 554	− 531	− 213	− 249	− 76	5	− 155	− 130	− 123	− 2046
*Percent 2019/2020*	3.7	4.3	− 8.1	− 28.3	− 25.7	− 11.2	− 12.1	− 4.9	0.3	− 7.5	− 6.5	− 6.1	− 8.7
*Difference 2019/2021*	− 103	− 25	− 226										− 354
*Percent 2019/2021*	− 5.2	− 1.4	− 10.7										
**Thorax (chest)**													
2019	**1290**	**1332**	**1554**	**1448**	**1562**	**1385**	**1536**	**1029**	**1426**	**1531**	**1320**	**1303**	16,716
2020	**1355**	**1396**	**1465**	**1033**	**1278**	**1310**	**1270**	**1048**	**1380**	**1490**	**1406**	**1471**	15,902
2021	**1342**	**1411**	**1452**										4205
*Difference 2019/2020*	65	64	− 89	− 415	− 284	− 75	− 266	19	− 46	− 41	86	168	− 814
*Percent 2019/2020*	5.0	4.8	− 5.7	− 28.7	− 18.2	− 5.4	− 17.3	1.8	− 3.2	− 2.7	6.5	12.9	− 4.9
*Difference 2019/2021*	52	79	− 102										29
*Percent 2019/2021*	4.0	5.9	− 6.6										
**Breast**													
2019	**7036**	**6178**	**7025**	**6314**	**6845**	**6551**	**7103**	**5261**	**6270**	**6645**	**6309**	**6162**	77,699
2020	**7203**	**6278**	**7150**	**5513**	**4241**	**5023**	**6343**	**5144**	**6712**	**6637**	**6977**	**6973**	74,194
2021	**7204**	**6555**	**7022**										20,781
*Difference 2019/2020*	167	100	125	− 801	− 2604	− 1528	− 760	− 117	442	− 8	668	811	− 3505
*Percent 2019/2020*	2.4	1.6	1.8	− 12.7	− 38.0	− 23.3	− 10.7	− 2.2	7.0	− 0.1	10.6	13.2	− 4.5
*Difference 2019/2021*	168	377	− 3										542
*Percent 2019/2021*	2.4	6.1	0.0										
**Ovary**													
2019	**609**	**631**	**727**	**626**	**672**	**635**	**714**	**484**	**664**	**723**	**626**	**662**	7773
2020	**593**	**642**	**691**	**492**	**549**	**686**	**708**	**576**	**668**	**675**	**644**	**684**	7608
2021	**598**	**671**	**662**										1931
*Difference 2019/2020*	− 16	11	− 36	− 134	− 123	51	− 6	92	4	− 48	18	22	− 165
*Percent 2019/2020*	− 2.6	1.7	− 5.0	− 21.4	− 18.3	8.0	− 0.8	19.0	0.6	− 6.6	2.9	3.3	− 2.1
*Difference 2019/2021*	− 11	40	− 65										− 36
*Percent 2019/2021*	− 1.8	6.3	− 8.9										
**Urology**													
2019	**3411**	**3474**	**3902**	**3527**	**3692**	**3539**	**3575**	**2217**	**3575**	**3894**	**3259**	**3348**	41,413
2020	**3594**	**3575**	**3546**	**2644**	**3408**	**3520**	**3147**	**2088**	**3449**	**3646**	**3689**	**3622**	39,928
2021	**3571**	**3616**	**3588**										10,775
*Difference 2019/2020*	183	101	− 356	− 883	− 284	− 19	− 428	− 129	− 126	− 248	430	274	− 1485
*Percent 2019/2020*	5.4	2.9	− 9.1	− 25.0	− 7.7	− 0.5	− 12.0	− 5.8	− 3.5	− 6.4	13.2	8.2	− 3.6
*Difference 2019/2021*	160	142	− 314										− 12
*Percent 2019/2021*	4.7	4.1	− 8.0										
**Overall**													
2019	**32,190**	**29,650**	**33,569**	**30,649**	**32,358**	**30,822**	**32,679**	**22,249**	**31,100**	**33,330**	**30,707**	**29,571**	368,874
2020	**33,000**	**30,679**	**30,127**	**20,370**	**23,671**	**28,733**	**30,346**	**21,996**	**31,896**	**31,456**	**32,246**	**31,207**	345,727
2021	**31,276**	**29,421**	**30,906**										91,603
*Difference 2019/2020*	810	1029	− 3442	− 10,279	− 8687	− 2089	− 2333	− 253	796	− 1874	1539	1636	− 23,147
*Percent 2019/2020*	2.5	3.5	− 10.3	− 33.5	− 26.8	− 6.8	− 7.1	− 1.1	2.6	− 5.6	5.0	5.5	− 6.3
*Difference 2019/2021*	− 914	− 229	− 2663										− 3806
*Percent 2019/2021*	− 2.8	− 0.8	− 7.9										

Number of excesions observed are in [bold].

For all excisions, the reduction in activity was very substantial, primarily in April, in which the whole month was spent under lockdown, in line with surgical activity cancellation directives. As such, for colorectal cancer excision, the shortfall was − 28,2% (− 902 procedures) in April, − 29.7% (− 938) in May, − 11.0% (− 318) in June, − 12.2% (− 421) in July, and − 1.6% (− 42) in August. For breast cancer, the greatest decrease was in May, − 38.0% (− 2604 procedures). The cumulative shortfall over the year came to 3505 surgical procedures, i.e. -4.5% despite greater activity in 2020 in the last months of the year.

For other cancer types, impacts on cancer excision are reported in Table 2.

### Chemotherapy (Table 3)

**Table 3:** Chemotherapy and radiotherapy activities.

	January	February	March	April	May	June	July	August	September	October	November	December	Total
**Chemotherapy − Stay and session**													
*2019*	**261,746**	**232,029**	**246,942**	**260,052**	**257,065**	**232,361**	**268,008**	**251,344**	**245,514**	**277,457**	**240,063**	**248,137**	3,020,718
*2020*	**269,722**	**240,690**	**250,771**	**240,149**	**227,922**	**251,182**	**262,722**	**239,286**	**257,318**	**260,488**	**252,180**	**272,040**	3,024,470
*2021*	**251,887**	**246,226**	**278,241**										776,354
*Difference 2019/2020*	7976	8661	3829	− 19,903	− 29,143	18,821	− 5286	− 12,058	11,804	− 16,969	12,117	23,903	3752
*Percent 2019/2020*	3.0	3.7	1.6	− 7.7	− 11.3	8.1	− 2.0	− 4.8	4.8	− 6.1	5.0	9.6	0.12
*Difference 2019/2021*	− 9859	14,197	31,299										−
*Percent 2019 − 2021*	− 3.8	6.1	12.7										−
**Chemotherapy − Persons**													
*2019*	**130,222**	**126,275**	**129,884**	**131,058**	**130,385**	**127,105**	**132,862**	**129,612**	**130,700**	**134,232**	**130,891**	**130,016**	323,394
*2020*	**135,370**	**132,347**	**133,626**	**125,052**	**128,920**	**132,364**	**134,565**	**131,316**	**135,563**	**137,184**	**137,370**	**139,049**	330,581
*2021*	**139,452**	**138,822**	**141,513**										186,927
*Difference 2019/2020*	5148	6072	3742	− 6006	− 1465	5259	1703	1704	4863	2952	6479	9033	7187
*Percent 2019/2020*	4.0	4.8	2.9	− 4.6	− 1.1	4.1	1.3	1.3	3.7	2.2	4.9	6.9	2.22
*Difference 2019/2021*	9230	12,547	11,629										−
*Percent 2019 − 2021*	7.1	9.9	9.0										−
**Radiotherapy − Inpatients**													
*2019*	**18,251**	**17,825**	**18,393**	**18,511**	**18,202**	**17,556**	**19,271**	**17,947**	**17,795**	**19,074**	**17,334**	**17,182**	114,930
*2020*	**18,115**	**17,549**	**17,576**	**15,420**	**15,353**	**17,652**	**17,805**	**15,730**	**16,440**	**16,990**	**16,603**	**16,922**	110,552
*2021*	**17,012**	**17,504**	**18,775**										36,250
*Difference 2019/2020*	− 136	− 276	− 817	− 3091	− 2849	96	− 1466	− 2217	− 1355	− 2084	− 731	− 260	− 4378
*Percent 2019/2020*	− 0.7	− 1.5	− 4.4	− 16.7	− 15.7	0.5	− 7.6	− 12.4	− 7.6	− 10.9	− 4.2	− 1.5	− 3.8
*Difference 2019/2021*	− 1239	− 321	382										−
*Percent 2019 − 2021*	− 6.8	− 1.8	2.1										−
**Radiotherapy − outpatients**													
*2019*	**17,944**	**16,652**	**17,340**	**17,308**	**17,105**	**16,563**	**18,352**	**15,967**	**16,567**	**17,780**	**16,191**	**16,678**	108,979
*2020*	**17,665**	**16,743**	**16,829**	**15,511**	**15,410**	**17,143**	**16,763**	**14,943**	**16,173**	**16,929**	**16,360**	**16,956**	107,384
*2021*	**16,410**	**15,622**	**15,001**										32,161
*Difference 2019/2020*	− 279	91	− 511	− 1797	− 1695	580	− 1589	− 1024	− 394	− 851	169	278	− 1595
*Percent 2019/2020*	− 1.6	0.5	− 2.9	− 10.4	− 9.9	3.5	− 8.7	− 6.4	− 2.4	− 4.8	1.0	1.7	− 1.5
*Difference 2019/2021*	− 1534	− 1030	− 2339										−
*Percent 2019 − 2021*	− 8.5	− 6.2	− 13.5										−

Numbers of persons, stays and sessions observed are in [bold].

The number of chemotherapy sessions was slightly up in January, February and March (+ 1.6%, 3,829 sessions/admissions), down − 7.7% (− 19,903) in April, and − 11.3% (− 29,143) in May, and subsequently fluctuated depending on the month. The number of patients treated over the year increased by 2.2% (+ 7,187).

### Radiotherapy (Table 3)

The number of patients receiving radiotherapy treatment dropped considerably both in the hospital sector and in private practice in April (− 16.7%, − 3091 patients, and − 10.4%, − 1797, respectively) and in May (− 15.7%, − 2849 and 9.9%, − 1695), and subsequently fluctuated depending on the month. Over the year, the decrease was 3.8% (− 4378 patients) in the hospital sector, and − 1.5% (− 1595) in private practice.

## Discussion

Regardless of the type of activity (diagnosis, screening and excision), the impact of the first lockdown is roughly the same: a shortfall in March, which worsened in April, partial recovery in May. The activity in the months following the easing of lockdown restrictions failed to bridge the gap observed, with activity reaching a similar level to the previous year.

Over the last months of the year, greater activity was observed in 2020 for mammograms, prostate biopsies, and excisions as a whole. Nevertheless, for oesophageal cancers and ENT cancers, the activity remained lower in 2020 compared to 2019 even at year end. Unlike the first lockdown, the restrictions applied from the end of October 2020 were not associated with a significant decrease in activity, suggesting a safeguarding of cancer care activity and the application of the guidelines issued following the first lockdown.

As such, over 2020, a substantial shortfall in activity remains, in terms of diagnosis, screening, and excisions. For the organised colorectal cancer screening programme, the results are deceptive, as the slight difference needs to be put into perspective with the disruptions in the supply of tests in 2019.

The main strength of this study is that it allowed regular monitoring of the health situation in cancer care based on exhaustive data, relating to prevention, screening, diagnosis, and care. The SNDS contains data from non-hospital settings and hospital data for the entire population. Although this medico-administrative database contains no clinical data, it enables effective monitoring of care activity. A further advantage of this study is that it covered a population of 67 million inhabitants, and included all patients regardless of their social or economic status or insurance scheme. Due to its exhaustive nature, it made it possible to monitor the health impact of the different lockdown periods, and of the different measures following the first lockdown and applied during subsequent months.

A number of lessons can be taken from these scorecards. During the first lockdown in March 2020, a substantial decline in activity as a whole was observed, both for the diagnostic and treatment activity. In the case of the latter, a lag occurred with a slight decrease in activity during March, but a later recovery came in June after the lockdown was lifted in May. This particularly reflects the impact of the health crisis on scheduled activity (particularly surgical activity). Furthermore, the shortfall in activity was not bridged in the months that followed the lifting of the lockdown. A further lesson relates to the lockdown initiated in November 2020. Unlike the first lockdown, the directives given to safeguard diagnostic and therapeutic activities in cancer care appear to have been followed, thus limiting the impact of this further lockdown period.

The impact of the health crisis is observed in many countries. For example, in the USA, several studies on medico-administrative databases report decreases of over 75% in breast, colorectal and cervical cancer screening activity during lockdown^12,18,19^. Similar findings are observed in Europe, with treatment-related activity also being impacted, with changes in practices and times to complete treatments^20–25^.

One limitation stems from the three-month time-frame for obtaining data, but scorecard processing is nonetheless not affected. Indeed, even though the aim is to enable accurate steering of the health crisis, these scorecards provide a good overview of the situation. This limitation is explained by the need to obtain exhaustive data. In the context of COVID-19, it is necessary to have daily monitoring of all hospitalisations due to COVID-19 in order to adapt health measures on a day-by-day basis. As regards cancer care activity monitoring, it is necessary to differentiate between a decrease in activity due to underreporting (lack of exhaustive data) and an actual decrease in activity. Furthermore, the measures adopted by the authorities on a national and local level and by healthcare professionals are measures to be applied over subsequent weeks or even months and do not require day-to-day steering.

Following on from these scorecards, further processing may be conducted to study the impact on a department or regional level. This could be compared to the spread of the virus in these areas. Besides these scorecards which do not allow monitoring of diagnostic and treatment activity for each individual, it would be beneficial to conduct follow-up studies on cancer patients’ care pathways. It will then be possible to estimate times to treatment, the potential transfer of activity particularly from surgery to chemotherapy and hormone therapy, or the forgoing of care. The number of chemotherapy inpatients is slightly higher in 2020, and there may be a carryover to oral therapies in non-hospital settings. The number of patients treated with radiotherapy is also lower, suggesting that the decrease in the number of sessions is not only due to the higher frequency of hypofractionated regimens. Follow-up studies will also help assess the impact of the current health crisis on recurrence and survival rates.

The primary causes identified during the first wave are the closure of so-called “non-emergency” medical activities, and low uptake of care, whether from primary care professionals or from cancer care professionals. According to a study by the Observatory on non-uptake of social rights and public services (Odenore) and the French national health insurance fund^26^, 60% of those surveyed report having decided not to receive one care that they needed at least once during the first lockdown. Similarly, 50% are of the view that not receiving care worsened their health problems.

It is difficult to obtain an accurate idea of the proportion attributable to these different causes of a shortfall in activity during the first wave and over time. Nevertheless, besides the waiting lists generated by the reduction in activity, non-uptake of care is likely to continue despite increased screening, diagnostic, and treatment capacities.

## Conclusion

Monitoring cancer care activity using SNDS data makes it possible to assess the impact of COVID-19 and the resulting health measures, particularly in the field of cancer care. The impact was greater in the first lockdown; however, despite the health measures adopted, the gap in activity does not appear to have been bridged. In some time, further studies will make it possible to assess the impact of COVID-19 not only on care pathways, recurrence, after-effects, and survival, but also on the forgoing of care.

## Data Availability

According to the European general data privacy regulation, an interested party interested in acceding data has to obtain an authorization from the French national ethic committee (CESREES) and the Cnil (French data protection authority) (contact: French national cancer institute).
